# *Asterarcys quadricellulare* (Chlorophyceae) protects H9c2 cardiomyoblasts from H_2_O_2_-induced oxidative stress

**DOI:** 10.1007/s11010-022-04626-7

**Published:** 2022-12-30

**Authors:** Imen Saadaoui, Touria Bounnit, Fatima Mraiche, Jensa M. Joseph, Maroua Cherif, Hareb Al-Jabri

**Affiliations:** 1 Centre for Sustainable Development, College of Arts and Sciences, Algal Technologies Program, Qatar University, 2713 Doha, Qatar; 2 Biological and Environmental Sciences Department, College of Arts and Sciences, Qatar University, 2713 Doha, Qatar; 3 Biomedical and Pharmaceutical Research Unit, QU Health, Qatar University, 2713 Doha, Qatar

**Keywords:** Antioxidant, *Asterarcys quadricellulare*, Cardiovascular diseases, Caspase-3, High value products, Reactive Oxygen Species

## Abstract

Oxidative stress has recently been identified as an important mediator of cardiovascular diseases. The need to find efficient antioxidant molecules is essential in the disease’s prevention. Therefore, the present study aimed to evaluate the potential of microalgae bioactive in protecting H9c2 cardiomyoblasts from H_2_O_2_-induced oxidative stress. Four microalgal species were investigated for their antioxidant capacity. A qualitative assessment of oxidative stress in H9c2 cardiomyoblasts stained with DCFH-DA, treated with the highly active microalgae extracts, was performed. The protein expression of total caspase-3 was also examined to investigate whether the extract protects H9c2 cardimyoblasts from H_2_O_2_-induced apoptosis. High antioxidant activity was observed for the hexanoic extracts after 10 days of cultivation. *Asterarcys quadricellulare* exhibited the highest antioxidant capacity of 110.59 ± 1.75 mg TE g^−1^ dry weight and was tested against H9c2 cardiomyoblasts, which were initially subjected to H_2_O_2_-induced oxidative stress. This hexanoic extract protected against H_2_O_2_ induced oxidative stress with a similar scavenging capacity as N-Acetylcysteine. Furthermore, total caspase-3 was increased following treatment with the hexanoic extract, suggesting that *A. quadricellulare* also had anti-apoptotic properties. The outcome of our study highlighted the possible use of the local *A. quadricellulare* strain QUCCCM10 as a natural, safe, and efficient antioxidant to prevent cardiovascular diseases.

## Introduction

Cardiovascular diseases (CVDs) are the number one cause of death globally, killing ~ 17.9 million annually. This represents 31% of total deaths worldwide (WHO, 2020). Previous studies provide comprehensive evidence that increased production of reactive oxygen species (ROS) is involved in the development and progression of cardiovascular diseases [1]. Several endogenous bioactive molecules with antioxidant capacity, such as vitamins and polyphenols, play a key role in ROS scavenging. Although highly efficient, these bioactives alone are not sufficient to protect, scavenge, and repair oxidative damage [2]. Therefore, serious cardiac tissue injury can take place if the delicate equilibrium between free radical production and antioxidant capability is altered [3].

External antioxidant sources are required to maintain such a balance and overcome oxidative stress. Therefore, consuming food enriched with bioactive molecules exhibiting high antioxidant capacity is highly recommended [4]. These antioxidants are mainly produced by photosynthetic organisms, and some of them are accumulated in terrestrial animal and marine ecosystems [2]. Various medicinal plants, seaweeds, and microalgae species have been investigated for their antioxidant potential for use as nutraceuticals and pharmaceuticals [5].

Carotenoids’ nutraceutical properties have piqued the interest of the food industry. Among all natural ones, five are considered to be the most economically important: β-carotene, Astaxanthin, Canthaxanthin, Lycopen and Lutein {Villa-Rivera, 2020 #56}. Numerous scientific studies have confirmed the health benefits of carotenoids, and their use for this purpose is increasing rapidly. However, finding a novel, safe, and viable source of antioxidants is still considered a valuable topic for research, since a renewable antioxidant source with optimized up- and down-stream processing for antioxidant production does not yet exist.

Microalgae are a very promising source of high value-added molecules with interesting biological activities, including but not limited to antibacterial, antiviral, antifungal, antiproliferative, and antioxidant activities [6, 7]. More importantly, some species of microalgae produce a panoply of primary phytochemicals with a significant role in the prevention of cardiovascular diseases (CVD) and strokes [8]. Different screening programs of microalgae isolates led to the identification and characterization of a wide range of bioactive molecules with high antioxidant potential, such as ascorbic acid, reduced glutathione, tocopherols, and carotenoids [9, 10].

Previous studies have proven that the production of carotenoids such high value chemicals (HVC) from algae can be enhanced by various physicochemical stresses [11]. As the Qatari climate poses several stress factors as high light intensities, temperatures, and salinities, it is expected that local microalgae isolates selected for the current study could present a very large bioactivity spectrum. To the best of our knowledge, our current research work is the first in vitro investigation of the impact of microalgae hexanoic extract using H9c2 cardiomyoblasts subjected to H_2_O_2_-induced oxidative stress. In this study, the microalgae crude extracts presenting the highest antioxidant capacity were tested for their ability to scavenge H_2_O_2_. The antioxidant capacity is compared to N-acetylcysteine [12], a conventional ROS scavenger. Finally, the impact of microalgae extracts on caspase-3 activation, a marker of apoptosis and mediator of cardiovascular disease, is also measured.

## Materials and methods

### Chemicals and materials

ABTS Antioxidant Assay Kit (Sigma-Aldrich, USA # CS0790) and 2,2-Diphenyl-1-(2,4,6-trinitrophenyl) hydrazyl were used to study antioxidant activity. Methanol (Fluka, # 34860) was used as an analytical standard to solubilize the bioactive molecules in the crude extracts. The antioxidant potential of the microalgae extracts was evaluated using N-acetyl L-cysteine (Sigma-Aldrich #A7250) [12]. The 2′7′-dichlorofluorescin-diacetate (DCFH-DA) (Sigma-Aldrich, #6883 SIGMA) was used to evaluate antioxidant scavenging capacity.

The standards used for the identification of the microalgae high value products are:

(i) astaxanthin (Sigma-Aldrich, P/N SML0982-50MG, Batch 86770), [1] beta-carotene (Sigma-Aldrich, P/N C9760-5G, Lot MKCK2908), (iii) canthaxanthin (Sigma-Aldrich, P/N 1175-1MG, Lot BCCB9917), and (iv) lutein (Sigma-Aldrich, P/N 07168-1MG, Lot BCCC1688).

### Culture of the microalgae strains

The strains used during this study belong to Qatar University culture collection of cyano-bacteria and microalgae (QUCCCM). Two freshwater microalgae strains: *A. quadricellulare* QUCCCM10; *N. conjuncta* QUCCCM28; and two marine species: *Chlorocystis* sp. QUCCCM60 and *P. atomus* QUCCCM130, were selected for the current study. These strains were subjected to investigation of their growth performance, metabolite contents and antioxidant capacities. Marine and freshwater microalgae strains were cultured in f/2 [13] and BG11 [14] growth medium, respectively.

For assessment of the growth properties and metabolite content, the microalgae strains were cultured as follows: One colony of each microalgae species was used to inoculate 10 ml of liquid growth medium before incubating at 30 °C with a 2 × g agitation, a photon flux density of 100 mol photons m^−2^ s^−1^, and 12 h:2 h dark: light cycles using an illuminated shaker, Innova® 44R incubator shaker, New Brunswick Scientific, https://www.eppendorf.com/. After 7 days of cultivation, the cultures were scaled up gradually to a volume of 100 ml and incubated under the same previously described conditions. Subsequently, an adequate volume was used to inoculate 1000 mL liquid growth medium with an initial optical density at 750 nm of 0.2 for the four strains. A daily assessment of the OD_750 nm_ was performed using a spectrophotometer Jenway 73100, http://www.jenway.com/. The biomass, harvested after 12 days of cultivation, was washed with water (freshwater algae) or ammonium formate 0.5 M (marine algae). It was then freeze-dried prior to metabolite extraction and quantification.

A different cultivation system targeting increase in producing high value products was applied. Cultures were scaled up gradually from 10 to 500 mL under the same previously described conditions. Then an adequate volume of this culture was used to inoculate a DASGIP parallel 1 L bioreactor system for phototrophic cultivation Eppendorf, https://www.eppendorf.com/ allowing higher light intensity than the shaker and better biomass productivity [15, 16]. This culture was grown for 20 days under a temperature of 30 °C, pH 8, an agitation of 2 × g, a continuous illumination of 400 μmol photons m^−2^ s^−1^ and 5% CO_2_, and using a depleted growth medium, (BG11 with 1/10 diluted nitrogen (NaNO_3_)). All cultures were performed in duplicate (*n* = 2). Samples were collected every 5 days (day 5, 10, 15 and 20), then washed and freeze-dried prior to being subjected to maceration for high value-added product extraction.

### Metabolites’ characterization of the microalgae isolates

For total protein, 25 mg of freeze-dried microalgae was dissolved in 5 mL of sodium hydroxide (NaOH 0.1 M) and incubated overnight at 60 °C. The total protein content was determined for the supernatant using the Folin-Ciocalteu reagent [17]. Serum albumin bovine was used as a standard.

Total lipids were extracted from the algae biomass using the same method as per Saadaoui et al. [15]. The total lipid content was determined gravimetrically. Subsequently, the lipid content (%) was determined as described using the following equations:

$$\% {\text{ Lipid content}}\, = \,\left( {{\text{Total Lipids }}\left( {\text{g}} \right)} \right)/\left( {{\text{Dry biomass }}\left( {\text{g}} \right)} \right)*{1}00$$

Total carbohydrates were extracted from 25 mg of freeze-dried microalgae using phenol sulfuric acid reagent biomass according to DuBois et al. [18]. Briefly, 25 mg was solubilized into glacial acetic acid, and incubated at 85 °C for 20 min to remove the chlorophyll. Then, the pellets were hydrolyzed using hydrochloric acid (HCl 4 M) at 90 °C for 2 h. Finally, the supernatant was neutralized with water and subjected to calorimetric assay using phenol sulfuric acid.

### Extraction and identification of bioactive molecules

The extraction of bioactive molecules was performed via maceration, using increasing polarity solvents such as hexane and acetone for both hydrophobic and hydrophilic metabolites, respectively. After 10 min of centrifuging at 2268×*g*, 20 mg of lyophilized microalgae was dissolved in 2 ml of organic solvent and incubated at 20 °C for 30 min. The supernatant was stored, and the pellet was dissolved again in 2 ml of organic solvent and incubated for 30 min at room temperature. This extraction was carried out twice to recuperate the microalgae extract. Finally, the pellet will be dissolved in 1% DMSO then stored at 4 °C in the obscurity for future use. The same protocol was used with both organic solvents.

For the identification of the high value products, the organic phase was separated, evaporated in a vacuum centrifuge, and reconstituted for injection on an ultra-high pressure liquid chromatography system coupled to an Orbitrap Fusion Lumos Tribrid Mass Spectrometer via an ultra-high-pressure liquid chromatography system. The analysis was carried out individually for positive and negative polarities, as well as for identification and quality control samples. The identification of the microalgae pigments was confirmed with the co-injection of 5 standards of β-carotene, lutein, astaxanthin, canthaxanthin, fucoxanthin. The quantification of the pigments was estimated using different concentrations of the standards.

### Evaluation of antioxidant activity

The level of antioxidant activity was determined for four selected strains extracted by acetone or hexane using the Trolox Equivalent Antioxidant Capacity assay (TEAC), utilizing the 2, 2’-Azino-Bis-3-Ethylbenzothiazoline-6-Sulfonic Acid (ABTS) antioxidant assay kit (Sigma-Aldrich kit, # CS0790, USA). The assay was performed as per the kit guidelines. Briefly, 6 concentrations of Trolox were used as a standard, and optic densities of 405 nm were determined via a synergy H4 hybrid multi-mode microplate reader Bio-Tek, https://www.biotek.com/. The decrease in absorbance reflected the ABTS + radical cation scavenging capacity and was plotted against the concentration of the antioxidant. The TEAC value represents the ratio between the slope of this linear plot for scavenging of ABTS + radical cation by the extract compared to the slope of this plot for ABTS + radical cation scavenging by Trolox, used as an antioxidant standard. TEAC was determined using the following formula:

$${\text{Antioxidant capacity }}\left( {\text{mM relative to the concentration of the Trolox standard}} \right)\, = \,\left( {{\text{Absorbance of the sample at 4}}0{5} {\text{nm}}\,{-}\,{\text{intercept}}} \right)/\left( {\text{slope of standard curve}} \right)*{\text{dilution factor}}$$

Antioxidant capacity was determined for all the hexane and acetone extracts at different times of harvesting (day 5, day 10, day 15, and day 20).

The extract presenting the highest antioxidant activity was selected to study its ROS scavenging potential in H9c2 cardiomyoblasts.

### Cell culture

H9c2 cardiomyoblasts, a clonal cell line derived from embryonic BD1 X rat ventricular heart tissue and known to exhibit many of the properties of skeletal muscle, were obtained from the American Type Culture Collection (ATCC). The H9c2 cell line was cultured in Dulbecco’s Modified Eagle Medium F12 1:1 (DMEM, Lonza Bioscience, United States), supplemented with 10% heat-inactivated Fetal Bovine Serum (FBS, Gibco, United States) and 1% Penicillin–Streptomycin (Gibco) in a CO_2_ incubator (5% CO2, 37 °C, Thermo Fisher Scientific, Waltham, MA, USA). To study the antioxidant activity, cells were treated with *A. quadricellulare* hexanoic extract (10 mg L^−1^) in the presence of H_2_O_2_ (100 μM). N-Acetylcysteine [12] (0.75 mM) and methanol were used as positive and negative controls, respectively.

### Qualitative assessment of oxidative stress in H9c2 cardiomyoblasts treated with microalgae extracts

Reactive oxygen species were directly visualized using H9c2 cardiomyoblasts stained with Dichloro-dihydro-fluorescein diacetate (DCFH-DA) and used as a qualitative assessment of the level of oxidative stress in H9c2 cardiomyoblasts which had been treated with the microalgae extracts presenting the highest antioxidant capacity. H9c2 cardiomyoblasts were plated in a microplate appropriate at a cell density of 500,000 cells and incubated for 24 h in a CO2 incubator Thermo Fisher Scientific, https://www.thermofisher.com/, 5% CO2, 37 °C. The growth medium was replaced by starvation media (0.5% FBS, 1% P/S) and incubated for 24 h in the presence of *A. quadricellulare* hexanoic extract (10 mg ml^−1^) and NAC separately. Then, cells were treated with H_2_O_2_ for 4 h. Cells incubated in the presence of *A. quadricellulare* hexanoic extract (10 mg ml^−1^) only and NAC, and H_2_O_2_ only, were used as control. In total, Five different treatments were applied as follows: (i) *A. quadricellulare* hexanoic extract (10 mg ml^−1^) alone; [19] *A. quadricellulare* hexanoic extract (10 mg ml^−1^) followed by H_2_O_2_ (100 mM); (iii) NAC (0.75 mM) alone; (iv) NAC (0.75 mM) followed by H_2_O_2_ (100 mM) and (v) H_2_O_2_ (100 mM) alone as negative control. 100 mM H_2_O_2_ was added for 4 h. Following treatment, the medium was aspirated, prior to being replaced by PBS and 10 µM DCFH-DA for 30 min. Following incubation with DCFH-DA for 30 min, the PBS dye was aspirated and replaced by only PBS, and images were taken using a Carl Zeiss Axiovert Fluorescence Inverted Microscope 40 CFL, https://bostonmicroscopes.com /. Cells with only PBS were used as negative controls, and cells with only H_2_O_2_ were used as positive controls. Fluorescence intensity (pixels) was estimated using ImageJ software.

### Apoptosis analysis by western blotting

Western blotting was used to detect the protein expression of total caspase-3. H9c2 cardiomyoblasts were treated as described above. Cells were then lysed using Radio Immunoprecipitation assay (RIPA buffer). Protein concentration was determined using the DC Protein assay kit (Bio-Rad). An equal amount of protein (15 μg) was loaded into 15% SDS-PAGE and transferred onto a nitrocellulose membrane. Eventually, the membranes were blocked with 5% Milk^−1^X TBST for 1 h, followed by overnight incubation at 4 °C with the following primary antibodies: caspase-3 (Santa Cruz, sc-271028). Anti-alpha tubulin (Abcam, ab4074) was used as the loading control. The membranes were then probed with horseradish peroxidase-conjugated secondary antibodies. Visualization for immunoreactivity was completed using enhanced chemiluminescence (ECL), followed by imaging and quantification of bands using the FCM MultiFluor System, https://www.proteinsimple.com/fluorchem.html.

### Statistical analysis

Data were expressed as the mean of two independent parallel experiments with two repetitions per experiment (a total of four values per variable). ANOVA was used to assess the differences among the treatments. The standard error of the means was calculated at < 0.05 level of significance.

## Results

### Morphology, growth, and metabolites characterization of the microalgae isolates

**Fig. 1:**
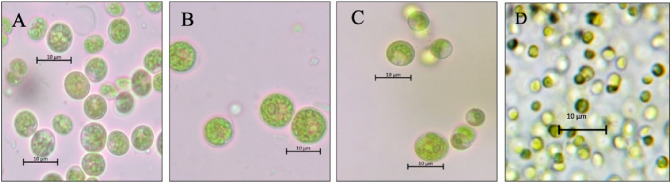
Morphological characterization of the microalgae isolates using light microscope with a magnification of 100X. **A**: *A. quadricellulare* QUCCCM10; **B**: *N. conjuncta* QUCCCM28; **C**: *Chlorocystis sp*. QUCCCM60 and **D**: *P. atomus* QUCCCM130.

Two freshwater and two marine microalgae were selected for the current study. The morphological characterization of these strains using a light microscope demonstrated that they are spherical in shape and have different sizes. QUCCCM28 and QUCCCM60 were the biggest in size at 10 μm, followed by QUCCCM10 (6 μm) then (2 μm) (Fig. 1). The growth rate determined for the four investigated strains was between 0.209 and 0.254 day-1 (Table 1). *N. conjuncta* QUCCCM28 presented the highest growth rate, while *A. quadricellulare* QUCCCM10 presented the lowest. We noticed that both marine strains presented almost the same growth rate of 0.24 per day^−1^. However, different metabolite contents were observed. The highest protein content was ob-served in the *P. atomus* QUCCCM130 and *A. quadricellulare* QUCCCM10 with ~ 33% g^−1^ dry weight while *N. conjuncta* QUCCCM28 presented the lowest protein content with 26% g^−1^ dry weight. All four strains presented interesting lipid content and the highest amount was observed in the case of *N. conjuncta* QUCCCM28 and *P. atomus* QUCCCM130 (~ 32% g^−1^ dry weight). Regarding the carbohydrate content, the highest amount was observed in the case of *A. quadricellulare* QUCCCM10 (19% g^−1^ dry weight) and the lowest was observed in the case of *P. maculatum* QUCCCM130 (6% g^−1^ dry weight).

**Table 1:** Growth performance and metabolites content of the selected microalgae isolates. All values recorded in triplicate (*n* = 3)

Microalgae isolate	Nature of the strain	μ (day^−1^)	Proteins %	Lipids %	Carbohydrates %
*A.quadricellulare* QUCCCM10	Freshwater	0.209	32.45 ± 0.02	25.0 ± 0.01	19.03 ± 0.07
*N. conjuncta* QUCCCM28	Freshwater	0.254	26.25 ± 0.01	30.5 ± 0.007	14.65 ± 0.12
*Chlorocystis* sp. QUCCCM60	Marine	0.244	31.50 ± 1.72	26.1 ± 0.62	ND^1^
*P. atomus* QUCCCM130	Marine	0.240	33.00 ± 0.02	32.7 ± 0.036	6.00 ± 0.001

*1ND* Not determined

### Antioxidant activity of microalgae isolates

**Fig. 2:**
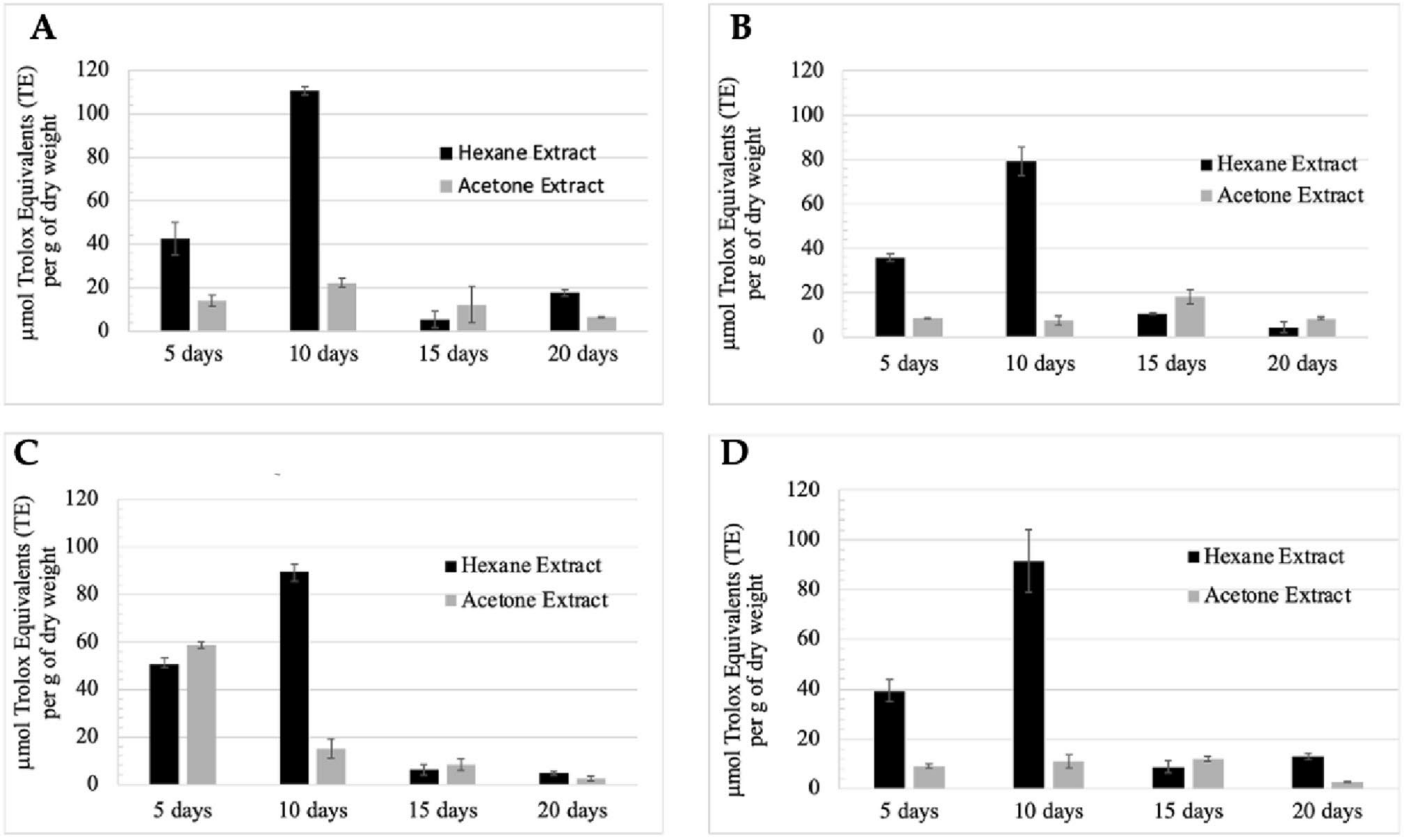
Antioxidant capacity of the microalgae crude extracts extracted using hexane and acetone. **A**: *A. quadricellulare;* **B**: *N. conjuncta* QUCCCM28; **C**: *Chlorocystis* sp. QUCCCM60 and **D**: *P. atomus* QUCCCM130. Measurements were recorded after 4-time intervals of microalgae growth, such as 5, 10, 15 and 20 days. All measurements were recorded as in triplicate (*n* = 3). The T-test was performed to study the significance of the differences (*p* < 0.05). The extract obtained after 10 days culture and showing maximum antioxidant capability

Hexanoic extracts showed higher antioxidant capacity than acetone extracts for the four selected strains collected at the four-time intervals investigated (5, 10, 15 and 20 days) (Fig. 2). This was proved by the significant difference observed (*p* < 0.05). There was only one exception corresponding to *Chlorocystis* sp. QUCCCM60, where the TEAC of the acetone extract (58.76 mg TE g^−1^ dry weight) was higher than the hexanoic extract (50.64 mg TE g^−1^ dry weight). The highest antioxidant capacity was observed at day 10 of cultivation. Hexanoic extracts from the biomass harvested after 10 days of cultivation showed a TEAC between 79.26 and 110.59 mg TE g^−1^ dry weight, while TEAC obtained for the acetone extracts was very low and varied between 10.91 and 15.23 mg TE g^−1^ dry weight. We also noticed that the average TEAC of fresh strains (94.92 mg TE g^−1^ dry weight) was higher than the average of the marine strains (90.58 mg TE g^−1^ dry weight). Among the four hexanoic extracts investigated, *A. quadricellulare* QUCCCM10 presented the highest antioxidant capacity with a TEAC of 110.59 ± 1.75 mg TE g^−1^ dry weight (*p* < 0.05), followed by *Chlorocystis* sp. QUCCCM60 and *P. atomus* QUCCCM130, both presenting a very similar TEAC of 89.6 ± 3.08 mg TE g^−1^ dry weight and 91.56 ± 12.5 mg TE g^−1^ dry weight, respectively. The lowest antioxidant capacity was recorded for the strain *N. conjuncta* QUCCCM28 with a TEAC of 79.26 ± 6.28 mg TE g^−1^ dry weight. Consequently, the hexanoic extract of the strain *A. quadricellulare* QUCCCM10, obtained after 10 days of cultivation, was used to investigate the impact of the bioactive molecules of this extract on the cardiomyoblast cells.

### Identification of the *A. quadricellulare* QUCCCM10’s bioactives

The spectral properties of the *A. quadricellulare* QUCCCM10 hexanoic extract were also investigated from 300 to 700 nm. The spectral scan proves the presence of a peak of absorbance at 420 nm, corresponding to the maximum absorbance of specific types of pigments such as carotenoids. The identification of the nature of the *A. quadricellulare* QUCCCM10 hexanoic extract pigments via GCMS-MS proved that it is mixture of the following carotenoids: Canthaxanthin (44%); Asthaxanthin (49%); B-carotenes, (2%), Lutein (5%).

### Morphological changes in H9c2 cardiomyoblasts treated with a hexanoic extract of QUCCCM10

**Fig. 3:**
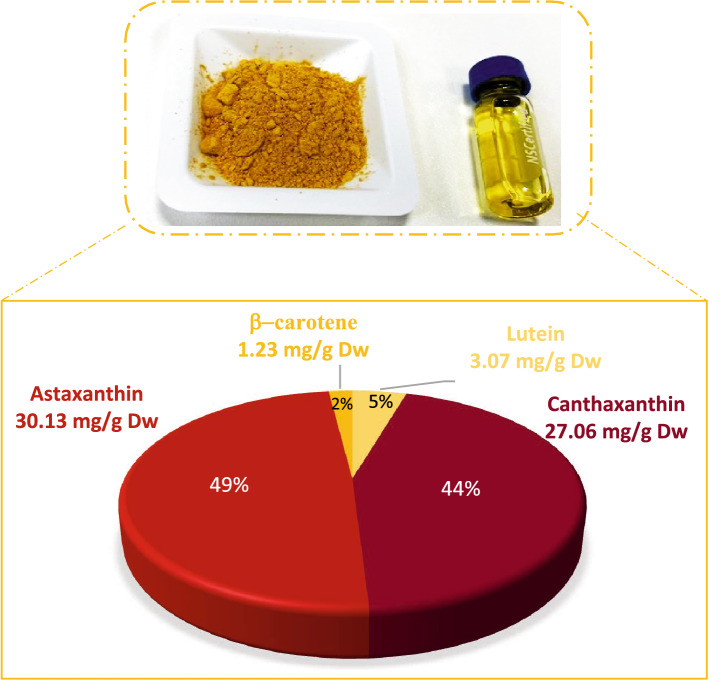
Identification of the Carotenoids Content of *A. quadricellulare* QUCCCM10 hexanoic extract obtained after 10 days culture and showing maximum antioxidant capability

**Fig. 4:**
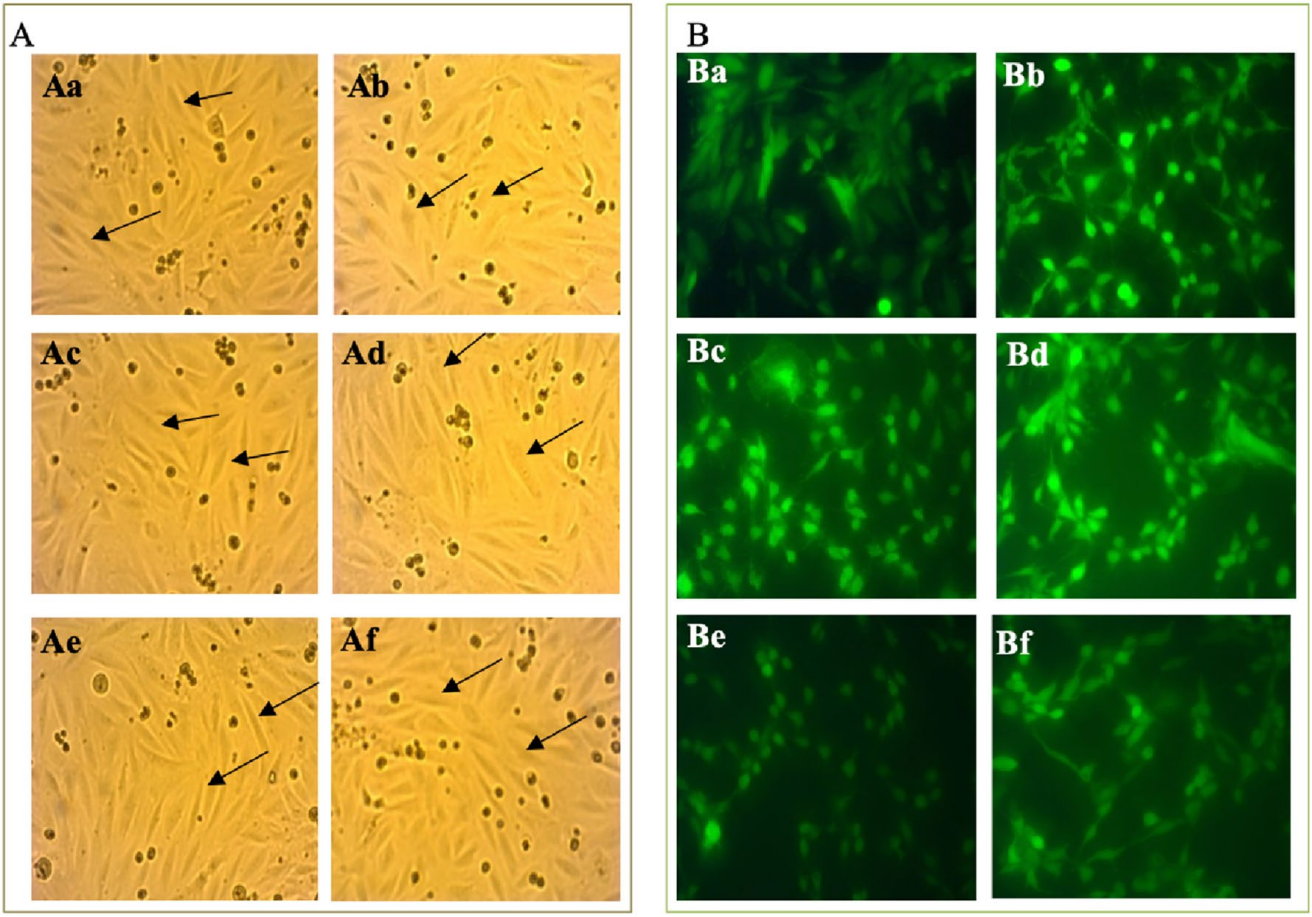
Changes in H9c2 cardiomyoblasts morphology following treatment with H_2_O_2_ in the presence of NAC or *A. quadricellulare* QUCCCM10 hexanoic extract obtained after 10 days culture and showing maximum antioxidant capability. **A**: Morphological Characterization via microscopic observation; **B**: fluorescence microscopy of H9c2 cardiomyoblasts stained with DCFH-DA and examined at magnification of 100X. H9c2 cardiomyoblasts were treated as following: Aa & Ba: without treatment (Control); Ab & Bb: treated with H202; Ac & Bc: *A. quadricellulare* QUCCCM10 hexanoic extract; Ad & Bd: NAC; Ae & Be: *A. quadricellulare* QUCCCM10 hexanoic extract and H_2_O_2_; Af & Bf: NAC and H202. N-Acetylcysteine

The morphology of the H9c2 cardiomyoblasts treated with H_2_O_2_ in the presence of *A. quadricellulare* QUCCCM10 hexanoic extract or NAC, a universal scavenger of ROS, was evaluated under a light microscope (Fig. 3A). The H9c2 cardiomyoblasts were normal; spindle-to stellate-shape [20] in the absence of H_2_O_2_. However, treatment with H_2_O_2_ resulted in cell shrinkage, and irregular shaping, as previously described [21] (Fig. 4A). Moreover, H9c2 cardiomyoblasts treated simultaneously with H_2_O_2_ and NAC showed the similar morphology as they are treated with H_2_O_2_ alone (Fig. 3Af), while *A. quadricellulare* QUCCCM10 hexanoic extract reversed the effects of H_2_O_2_ (Fig. 3Ae)_._

The antioxidant potential of *A. quadricellulare* QUCCCM10 hexanoic extracts was examined in H9c2 cardiomyoblasts stained with DCFH-DA [22]. Staining of H9c2 cardiomyoblasts with DCFH-DA demonstrated that H_2_O_2_ resulted in greater fluorescence than the control. Treatment with NAC or *A. quadricellulare* QUCCCM10 hexanoic extract in the presence of H_2_O_2_ resulted in less florescence than H_2_O_2_ alone, suggesting that *A. quadricellulare* QUCCCM10 hexanoic extract scavenges ROS. The effect appears to be more pronounced in the presence of *A. quadricellulare* QUCCCM10 with evidencing a fluorescence intensity of 21 ± 1.66 pixels compared to the 25 ± 1.30 pixels observed in the case of cells treated with H_2_O_2_ and NAC. The difference between both fluorescence intensities appeared as significative (*p* < 0.05). Our results suggest the potential of *A. quadricellulare* QUCCCM10 in scavenging ROS and preventing H9c2 from damage associated with oxidative stress (Fig. 4B).

### *A. quadricellulare* QUCCCM10 altered caspase-3 activity in H9c2 cardiomyoblasts treated with H202

**Fig. 5:**
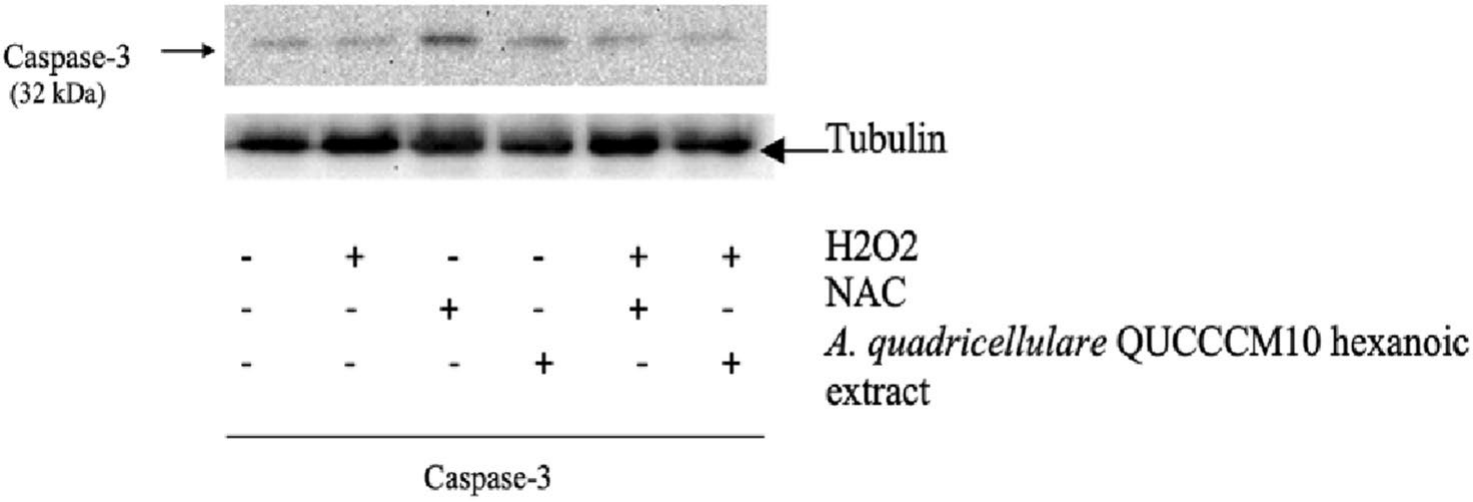
The effect of *A. quadricellulare* QUCCCM10 hexanoic extract, obtained after 10 days culture and showing maximum antioxidant capability, on total caspase-3 protein expression in H9c2 cardiomyoblasts subjected to H_2_O_2_. Representative western blot of total caspase in H9c2 cardiomyoblasts treated with H_2_O_2_ in the presence and absence of *A. quadricellulare* QUCCCM10 hexanoic extract or NAC. Equal amount of proteins was loaded

Caspase-3, as an effector enzyme, plays a key role in promoting apoptosis [23]. The protein expression of total caspase-3 was examined by western blot analysis to investigate whether *A. quadricellulare* QUCCCM10 hexanoic extract protects H9c2 cardimyoblasts from H_2_O_2_-induced apoptosis (Fig. 5). Total caspase-3 protein expression was decreased in H9c2 cardiomyocytes in the presence of H_2_O_2_. Co-treatment of H9c2 cardiomyoblasts with *A. quadricellulare* QUCCCM10 increased total caspase-3 expression.

## Discussion

The unique biodiversity of Qatar marine environment provides a large source of novel and bioactive molecules from microalgae. To survive the long-term unfavorable environmental conditions faced in the desert environment, local microalgae establish an antioxidant defense system as a survival mechanism. Among these compounds, carotenoids can combat the oxidative damage caused by biotic or abiotic stressors by scavenging reactive oxygen species (ROS) [24].

The idea of this work revolves on the exploitation of novel microalgae as a source of bioactive molecules against CVD. Previous studies have focused on biomolecules from microalgae belonging to different ecosystems, however in this research native microalgal strains adapted to the harsh climate are considered.

The obtained results demonstrated that antioxidant activity was variable depending on the species and the organic solvent used for the extraction. The algae extract obtained using acetone did not show high antioxidant activity compared to those extracted using hexane. Hexane is known for its efficiency in extracting hydrophobic compounds [25]. Moreover, it is demonstrated that the yield and nature of bioactive compounds mainly depends on the polarity of the solvent and the chemical properties of the sample, with polar solvents considered more efficient than non-polar [26]. All the extracts of the investigated algal species demonstrated promising antioxidant activity with *A. quadricellulare* QUCCCM10 as the highest for the freshwater strain and *Chlorocystis* sp. QUCCCM60 and *P. atomus* QUCCCM130 for the marine isolates. A previous study reported that samples with a TEAC of activity more than 10 μmol Trolox eq. g^−1^ DW are considered high in antioxidant content [27]. The lowest antioxidant capacity was recorded for the strain *N. conjuncta* QUCCCM28 with a TEAC of 79.26 ± 6.28 mg TE g^−1^ dry weight. Furthermore, the highest antioxidant capacity was detected after 10 days for the four different strains belonging to different species, suggesting that the production of antioxidant agents is dependent on the growth stage and not on the microalgae species.

The spectral scan of the *A. quadricellulare* QUCCCM10 hexane extract revealed a peak of absorbency at 420 nm proving that carotenoids are the major compound of the extract. This peak was observed under continuous light and repleted nitrogen. Such result converged with Aydi et al., [28] findings stating that carotenoids are synthesized by algae in response to continuous light, nitrate limitation. Furthermore, the hexane crude extracted presenting the highest antioxidant activity was obtained from a biomass collected during the stationary phase (cultured for 10 days) and this phase is characterized by the accumulation of these molecules [29]. Such results join the findings of Guedes et al., [30], stating that the antioxidant power of algae comes essentially from carotenoids that are produced at the stationary phase [31]. This agrees with previous research work that highlighted the same findings where the maximum of the carotenoids production for the strain *A. quadricellulare* (25 mg/g dry wt biomass) responsible for the antioxidant activity studied was observed at the stationary phase under unfavorable conditions [32]. More importantly, the total carotenoids content of *A. quadricellulare* QUCCCM10 reaching 61.5 mg/g dry weight is ~ 2.5 × higher that the findings reported by Singh et al., [32].

The pigments found in the hexane extract of *A. quadricellulare* QUCCCM10 were identified as astaxanthin (49%) canthaxanthin (44%) lutein (5%), and β-carotene (2%). The primary pigments are astaxanthin and canthaxanthin, which account for 93% of the total pigments. Both pigments had a total concentration of 57.19 mg/g dry Wt when combined.

Goiris et al. [33] found that extracts from *Chlorella vulgaris* had strong antioxidant activity due to their high content of bioactive compounds such as carotenes, astaxanthin, lutein, and fucoxanthin. Rodriguez-Garcia and Guil-Guerrero [12] both reported the same information. Another research on *Neochloris oleoabundans* extracts found that, if given the right growing circumstances, these green microalgae may create significant levels of carotenoids, which aid in the prevention of CVD [34].

Previous research work stated that consumption of a diet high in astaxanthin and canthaxanthin, also called the “kings of carotenoids” showed to lower blood pressure, delay atherogenesis, and improve cardiovascular parameters by reducing oxidative stress and increasing nitrogen oxide bioavailability [35, 36]. Moreover, Di Pietro et al., studied the association of carotenoids (β -carotene, lycopene, lutein, zeaxanthin, and -cryptoxanthin) with the risk of cardiovascular disease and atherosclerosis and concluded that there is a positive relationship between a higher intake of carotenoids rich fruits and vegetables and a lower risk of morbidity and mortality from cardiovascular disease [36]. This effect was primarily due to redox balance protection that carotenoids can provide. Likewise, a high dietary intake by pregnant women of one of the most common carotenoids found in nature, β-carotene, reduced the incidence of CVD in particular the risk of nonfatal acute myocardial infarction [36]. Moreover, it was reported that a low level of β-carotene in the blood was generally associated with an increased risk of sudden cardiac death and ischaemic heart disease [37]. Recently, lutein has emerged as a potent candidate for atheroprotection [38] and studies revealed the long-term benefit of lutein supplements on cardiovascular health of 74 years old people [39].

As per the literature, many scientific studies have confirmed the benefits of the four identified carotenoids to health. They can contribute individually as membrane antioxidants, thus participating in the oxidative cell cycle, or together in the control processes of cell differentiation and proliferation [40]. Palozza et al. investigated the antioxidant activity of some carotenoids during radical peroxide-induced cholesterol oxidation, and they reported significant antioxidant activity of carotenoids in the decreasing activity order indicated: astaxanthin, cantaxanthin, lutein, and –carotene [41], with astaxanthin displaying 10 times stronger antioxidant activity than other carotenoids and 550 times the potency of alpha tocopherol as a singlet oxygen quencher [42].

Thus, the majority of *A. quadricellulare* QUCCCM 10’s antioxidant capacity can be reported for canthaxanthin and its keto-carotenoid derivative, astaxanthin which play a play a key role in free radical scavenging [43]. Astaxanthin’s high potency and polar characteristics make it an appealing nutraceutical for future research in atherosclerotic cardiovascular disease, where antioxidant cellular protection might be beneficial Canthaxanthin, on the other hand, has been linked to antioxidant and apoptotic properties [43].

The morphology of the H9c2 cardiomyoblasts treated with H_2_O_2_ demonstrated that the H_2_O_2_ affected the cell morphology of the strains and created more visible changes and hypertrophy. This converges with Branco et al. [21] which suggests that ROS modifies cell shape and size. However, *A. quadricellulare* hexanoic extract maintained the morphology of the cardiomyoblasts subjected to H_2_O_2_. Thus, suggesting that *A. quadricellulare* protects H9c2 cardiomyoblasts from oxidative damage, an effect which is comparable to that of NAC. NAC is a thiol-containing antioxidant that modulates the intracellular redox state. NAC can scavenge reactive oxygen species (ROS) to protect cardiomyocytes from oxidative stress [44].

Furthermore, *A. quadricellulare* hexanoic extract was demonstrated to have efficient scavenging capacity, which was demonstrated using DCFH-DA, the most widely used fluorometric probe for detecting intracellular oxidative stress [45]. Results confirmed that microalgae bioactives can play a crucial role in preventing human disease caused by oxidative stress as it was previously stated [46]. Indeed, carotenoids have been shown to reduce the risk of cardiovascular disease induced by reactive nitrogen species [47].

We also found that total caspase-3, which contributes to DNA fragmentation, leads to apoptosis and plays an important role in CVD, was increased following treatment with *A. quadricellulare* QUCCCM10 hexanoic extract. Our findings of reduced caspase-3 activity following stimulation with H202 agree with an earlier study [48]. The ability of algae to reverse this decrease in total caspase-3 support the suggestion that QUCCCM10 hexanoic extract has anti-apoptotic effects. Further studies are needed to assess this potentially promising algae extract in vivo and in the clinic.

## Conclusions

The antioxidant activity is dependent on the species and the organic solvent used for the extraction. However, the bioactives’ production is maximal at the stationary phase. *A. quadricellulare* QUCCCM10 hexanoic exhibited the highest antioxidant capacity. This extract protected H9c2 cardiomyoblasts against H_2_O_2_ induced oxidative stress with similar scavenging capacity as NAC. Furthermore, total caspase-3, was increased following treatment with the hexanoic extract, suggesting that *A. quadricellulare* QUCCCM10 also had anti-apoptotic properties. The current study presented *A. quadricellulare* as natural, safe, and efficient source of antioxidant to prevent cardiovascular disease.

## Data Availability

Data are available on request to the authors.
